# Brain network involved in visual processing of movement stimuli used in upper limb robotic training: an fMRI study

**DOI:** 10.1186/1743-0003-9-49

**Published:** 2012-07-24

**Authors:** Federico Nocchi, Simone Gazzellini, Carmela Grisolia, Maurizio Petrarca, Vittorio Cannatà, Paolo Cappa, Tommaso D’Alessio, Enrico Castelli

**Affiliations:** 1 Clinical Technology Innovations Research Area, Bambino Gesù Children’s Hospital, IRCCS, Piazza S. Onofrio 4, Rome, Italy; 2 Department of Applied Electronics, University Roma Tre, Via della Vasca Navale 84, Rome, Italy; 3 Department of Neuroscience and Neurorehabilitation, Bambino Gesù Children’s Hospital, IRCCS, Via Torre di Palidoro, Passoscuro, Rome, Italy; 4 MARlab (Movement Analysis and Robotics Laboratory), Neurorehabilitation Division of Bambino Gesù Children’s Hospital, IRCCS, Via Torre di Palidoro, Passoscuro, Rome, Italy; 5 Department of Mechanical and Aerospace Engineering, Sapienza University of Rome, Via Eudossiana 4, Rome, Italy

**Keywords:** Neurorehabilitation, Robot-mediated therapy, fMRI, Motor processing, Upper limb rehabilitation

## Abstract

**Background:**

The potential of robot-mediated therapy and virtual reality in neurorehabilitation is becoming of increasing importance. However, there is limited information, using neuroimaging, on the neural networks involved in training with these technologies. This study was intended to detect the brain network involved in the visual processing of movement during robotic training. The main aim was to investigate the existence of a common cerebral network able to assimilate biological (human upper limb) and non-biological (abstract object) movements, hence testing the suitability of the visual non-biological feedback provided by the InMotion2 Robot.

**Methods:**

A visual functional Magnetic Resonance Imaging (fMRI) task was administered to 22 healthy subjects. The task required observation and retrieval of motor gestures and of the visual feedback used in robotic training. Functional activations of both biological and non-biological movements were examined to identify areas activated in both conditions, along with differential activity in upper limb vs. abstract object trials. Control of response was also tested by administering trials with congruent and incongruent reaching movements.

**Results:**

The observation of upper limb and abstract object movements elicited similar patterns of activations according to a caudo-rostral pathway for the visual processing of movements (including specific areas of the occipital, temporal, parietal, and frontal lobes). Similarly, overlapping activations were found for the subsequent retrieval of the observed movement. Furthermore, activations of frontal cortical areas were associated with congruent trials more than with the incongruent ones.

**Conclusions:**

This study identified the neural pathway associated with visual processing of movement stimuli used in upper limb robot-mediated training and investigated the brain’s ability to assimilate abstract object movements with human motor gestures. In both conditions, activations were elicited in cerebral areas involved in visual perception, sensory integration, recognition of movement, re-mapping on the somatosensory and motor cortex, storage in memory, and response control. Results from the congruent vs. incongruent trials revealed greater activity for the former condition than the latter in a network including cingulate cortex, right inferior and middle frontal gyrus that are involved in the go-signal and in decision control. Results on healthy subjects would suggest the appropriateness of an abstract visual feedback provided during motor training. The task contributes to highlight the potential of fMRI in improving the understanding of visual motor processes and may also be useful in detecting brain reorganisation during training.

## Background

New technologies such as robotics and virtual reality are increasingly being used alongside traditional rehabilitation treatments in order to assist, enhance and assess motor training. The role of these technologies within therapeutic treatment programs and their effectiveness are currently under debate [1-6]. In particular, their association with traditional therapy [7] and the differences between improvements in motor control and in activities of daily living are discussed in the literature [8]. Recent evidence suggests that the contribution of new technologies to clinical practice is currently limited to providing intensive and repetitive movements and that the potential of robotic devices in neurorehabilitation has not been completely investigated [9]. Furthermore, there is limited description of the neural networks involved and of the brain reorganisation related to training. In this regard, neuroimaging techniques represent a powerful tool. Simple and complex movement and motor imagery tasks were used to investigate motor processing in healthy and neurologically impaired subjects in experiments with functional Magnetic Resonance Imaging (fMRI) [10-12], Single Photon Emission Computerised Tomography [13], Positron Emission Tomography [14-16], Magnetoencephalography [17], and Direct Current potentials [18,19]. Moreover, changes have been described in neuronal motor networks as a consequence of a rehabilitation training by means of functional and morphometric Magnetic Resonance techniques [20-23].

In studies with fMRI involving patients suffering from motor deficits, the selection of an appropriate task is a particularly relevant issue [24] and visual tasks may be preferred to motor ones in order to reduce involuntary head movements during scanning. With regard to this aspect, it has been shown that the neural network activated in humans following the observation of an action overlaps with that involved during the execution of the same action [25-27]. Furthermore, recent clinical trials have proved that action observation in chronic stroke patients improves functional abilities measured by clinical scales [28,29] and induces cortical reorganisation as revealed by enlarged fMRI activation in the bilateral ventral premotor cortex, bilateral superior temporal gyrus, supplementary motor area (SMA) and contralateral supramarginal gyrus [28].

However, there is an open question regarding whether or not the processing of non-biological movements relies on the same structures as those involved in biological motion processing. In case of light-points portraying human biological motion significant fMRI activations were reported, compared with non-rigid random points motion, in the lingual and fusiform gyri, superior temporal sulcus (STS), kinetic occipital area (KO, lateral occipital cortex sensitive to kinetic contours), and lateral cerebellum [30]. Servos *et al.*[31] confirmed the role of the lingual gyrus during the recognition of light-points simulating human biological motion. The specific involvement in the perception of biological movements of the right posterior STS has also been highlighted by previous studies [32,33]. Further data support the need of observing biological plausible movements to elicit a corresponding pre-motor cortex activation [34,35]. On the contrary, other works reported that the observation of upper limb movements carried out by humanoid robotic devices may generate the same fMRI pattern activated by the observation of the human arm [36-38]. Saygin *et al.*[39] reported no difference in the fMRI activation pattern during observation of body movements executed by humans or by robots. However, they found that the anterior part of the intraparietal sulcus (IPS) was significantly active in detecting the mismatch between appearance and motion, as in the case of androids with biological appearance performing mechanical movements.

**Figure 1:**
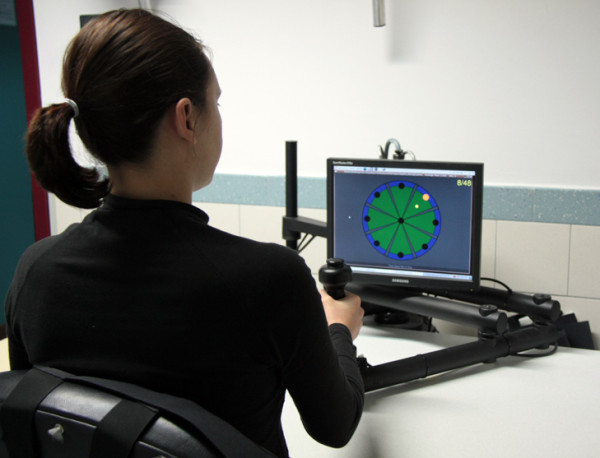
**InMotion2 robotic setting**.

In this study, we present a visual fMRI task able to indirectly estimate, by measuring brain haemodynamics [40,41], the neuronal activation patterns related to the visual stimulation during upper limb robotic training. The task simulates the human and abstract object movements observed during a planar point-to-point reaching task with the InMotion2 Robot (Interactive Motion Technologies Inc., Boston, Massachusetts, USA), a device that exploits both visual and haptic feedback [42] (Figure 1). Human movements are presented as an arm performing a reaching gesture similar to that carried out during robotic training, whereas abstract object movements consist in a dot moving along straight trajectories that represent the visual feedback provided by the InMotion2 Robot. The experimental task was designed to identify the cerebral activity associated with visual processing (observation, analysis, and representation) of movements similar to those executed during robotic training. The main aim was to verify commonalities and differences in the brain networks able to process biological (human upper limb) and non-biological (abstract object) movements. A group of healthy subjects participated in this study. Comparing neuronal activity before and after rehabilitative training in impaired subjects is out of the scope of the present study. However, this analysis could be useful in testing the biological effectiveness of the visual feedback associated with robotic training. Furthermore, this study could contribute to the current debate about the use of action observation training in the treatment of stroke and cerebral palsy [43,44].

## Methods

### Subjects

The study was carried out on a sample of 22 healthy volunteers (8 males, age 25.6 ± 4.3 years, min = 19.2, max = 36.0). The inclusion criteria were: 18 to 39 years of age; right-handedness; no current or previous motor and neurological disorders; no current or previous rehabilitation protocols or robot-mediated training. A clinical evaluation was performed in each participant by a skilled physician prior to scanning. Informed consent was obtained from all subjects and the study was approved by the hospital’s Ethical Committee.

### Experimental design

**Figure 2:**
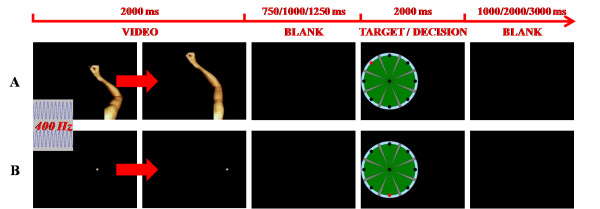
**Schematic representation of the fMRI task.** Human arm planar movements (**A**) and straight trajectories of a dot (**B**), are followed by a representation of the video screen presented during the rehabilitation training with the InMotion2 Robot, with a randomly positioned target (red dot). Subjects were asked to compare the direction of each motion stimulus with the position of the target. A congruent (**A**) and an incongruent (**B**) trial are shown.

An fMRI visual task was set up. The task consisted in administering a video sequence of both human arm planar movements and straight trajectories of a dot, presented in a random order, along eight possible directions starting from the centre of the screen. The motion stimuli were followed by the image of the video screen presented during the rehabilitation training with the InMotion2 Robot, with a randomly positioned target point. The subjects were required to compare the direction of the observed motion stimulus (arm planar movement or dot trajectory) with the target position (Figure 2) and they were asked to mentally count the number of congruencies of the target position with any kind of motion stimulus. We hypothesised that, in each trial, the subject should process the observed stimulus (arm movement or dot trajectory), retrieve the arm movement or the dot trajectory, assess the congruence between direction of the stimulus and position of the target, and make a decision. According to this hypothesis, arm and dot trials only differ in the perceptual analysis of the anthropomorphic movement, which is absent in the case of the dot trajectory presentation. The use of a visual task allowed reducing involuntary head movements during the fMRI session. With the same aim, any kind of motor response from the subjects (e.g.: pushing a response button in a keypad) was avoided.

Both right and left arm movements from a healthy adult subject were video recorded during the execution of an InMotion2 training session (planar point-to-point reaching task) and adapted for the stimulation sequence. An acoustic signal was given to the subjects at the beginning of each trial (i.e., when an arm movement or a dot trajectory started). A time interval of random duration (750, 1,000, or 1,250 ms), during which subjects saw a blank screen, followed the motion stimulus and preceded the presentation of the target. A second variable time interval (1,000, 2,000, or 3,000 ms) was introduced following the target and before the onset of the successive motion stimulus (Figure 2). Four runs were performed by each subject. Each run consisted of 54 trials with randomised presentation of both biological and non-biological stimuli and all stimuli (right arm movements, left arm movements and dot trajectories) occurring with the same frequency at each run. Taking the 4 runs together, each movement direction was presented the same number of times and an equal number of congruent and incongruent trials occurred. To avoid possible ambiguities related to the interpretation of the human arm movements, the angular mismatch between the movement direction and the position of the target was greater than or equal to 90° both for arm movements and dot trajectories.

A 15’ practice session with the InMotion2 Robot was administered to each subject one hour before the fMRI exam. This very short session, aimed at familiarising with the task used in fMRI, was part of the instructions given to subjects prior to scanning and was followed by administering a set of trials of the fMRI task to verify their ability to perform it.

### MRI acquisition

All scans were performed on the same MRI scanner (1.5 T Achieva, Philips Medical Systems, Best, the Netherlands) equipped with an 8-channels Sense head coil. The imaging protocol for each subject consisted of a 3D T1-weighted anatomical scan acquired as a structural reference and of 4 T2*-weighted Echo-Planar Imaging (EPI) sequences. The following parameters were used in EPI scans: repetition time (TR) = 2,500 ms, echo time (TE) = 46 ms, flip angle = 90°, field of view (FOV) = 256x140x256 mm, 28 axial slices (slice thickness = 5 mm, without gap), reconstruction matrix = 64x64 (pixel size = 4x4 mm), 6 dummy scans and 153 dynamic scans for each sequence (duration: 6’22”). The total scan time for the protocol was 31’45”. The stimuli sequences were implemented in the STIM2 software (Compumedics Neuroscan, El Paso, Texas, USA) and delivered by an MR-compatible stimulation system (NordicNeuroLab, Bergen, Norway).

### FMRI data analysis

Analysis of fMRI data was performed with SPM8 (The Wellcome Department of Imaging Neuroscience, University College, London, UK) running in MATLAB (version 7.9.0.529, R2009b) (The Mathworks, Natick, Massachusetts, USA). For each subject, after motion correction, the anatomical MRI was coregistered to the mean functional image. Parameters for normalisation to the standard Montréal Neurological Institute (MNI) space were subsequently estimated and applied to the functional images. Finally, spatial smoothing was performed by convolving with an 8-mm full-width at half-maximum isotropic Gaussian kernel.

Statistical analysis was performed in a 2-stage mixed-effect procedure using the general linear model approach for event-related fMRI designs. In the 1^st^ level, individual subject analysis, 6 regressors were used in each run to model the blood oxygenation level dependent (BOLD) response for each of 6 event types. The events were defined as observation of an arm movement (AM), observation of a dot trajectory (DT), presentation of a congruent target point following an AM (AMCT), presentation of a congruent target point following a DT (DTCT) and presentation of incongruent target points following AMs or DTs (AMIT and DTIT, respectively). Estimates of the 6 head movement parameters obtained from the realignment stage of pre-processing were included as additional regressors. Contrasts between regressors were then obtained for each subject.

The results from the 1^st^ level analysis were entered into an one-sample t-test for the 2^nd^ level analysis, thus enabling inferences based on the contrasts to be extended to the population from which the subjects were drawn [45]. All statistical parametric maps (SPMs) were thresholded at p < 0.001 at the voxel-level (uncorrected) and only clusters surviving a family-wise error (FWE) corrected threshold of p < 0.05 were considered significant [46]. A conjunction analysis of AM vs. implicit baseline (IB) and DT vs. IB contrasts was performed to identify areas activated in both conditions. The t-maps of these contrasts at the group level were thresholded, binarised, and multiplied voxel-wise with each other [47]. The analysis of congruence between the observed arm movement or dot trajectory and the position of the target point implies retrieval from memory of the previously observed movement and imagery of the movement toward the target, which were referred to as “representation of arm movements” (AMT) and “representation of dot trajectories” (DTT). A second conjunction analysis was performed for these “representations”.

## Results

### Subjects’ performance

At the end of each fMRI run, subjects reported the number of congruent trials. Errors were less than 12 % for each run in each subject. This low percentage error was assumed to be a sufficient proof of subjects’ continued participation in the task. Therefore, no subject nor run was excluded from the group analysis due to low task performance.

### Observation of arm movements and dot trajectories

When the observation of AMs was compared to the IB, a significant bilateral activation was found in the occipital lobe (primary, secondary and associative visual cortex, Brodmann Areas (BA) 17, 18, 19), parietal lobe (superior parietal lobules, BA 5, 7; supramarginal gyrus, BA 40; angular gyrus, BA 39; postcentral gyrus, BA 3, 1, 2; precuneus, BA 7 medial; paracentral lobule, BA 3, 1, 2, 5 medial), temporal lobe (middle and inferior temporal gyrus, fusiform gyrus, BA 20, 21, 22, 37), several areas of the frontal lobe (precentral gyrus-primary motor area, BA 4; SMA, BA 6; medial frontal gyrus, BA 8, 9; superior frontal gyrus, BA 4, 6, 8; middle frontal gyrus, BA 9; inferior frontal gyrus, BA 44, 46, 47), limbic lobe (cingulate gyrus, BA 31; hippocampus and parahippocampal gyrus, BA 27, 30), and insula (BA 13). Furthermore, activations were found in a large portion of the cerebellum (vermis, anterior and posterior cortex) and in the thalamus.

**Figure 3:**
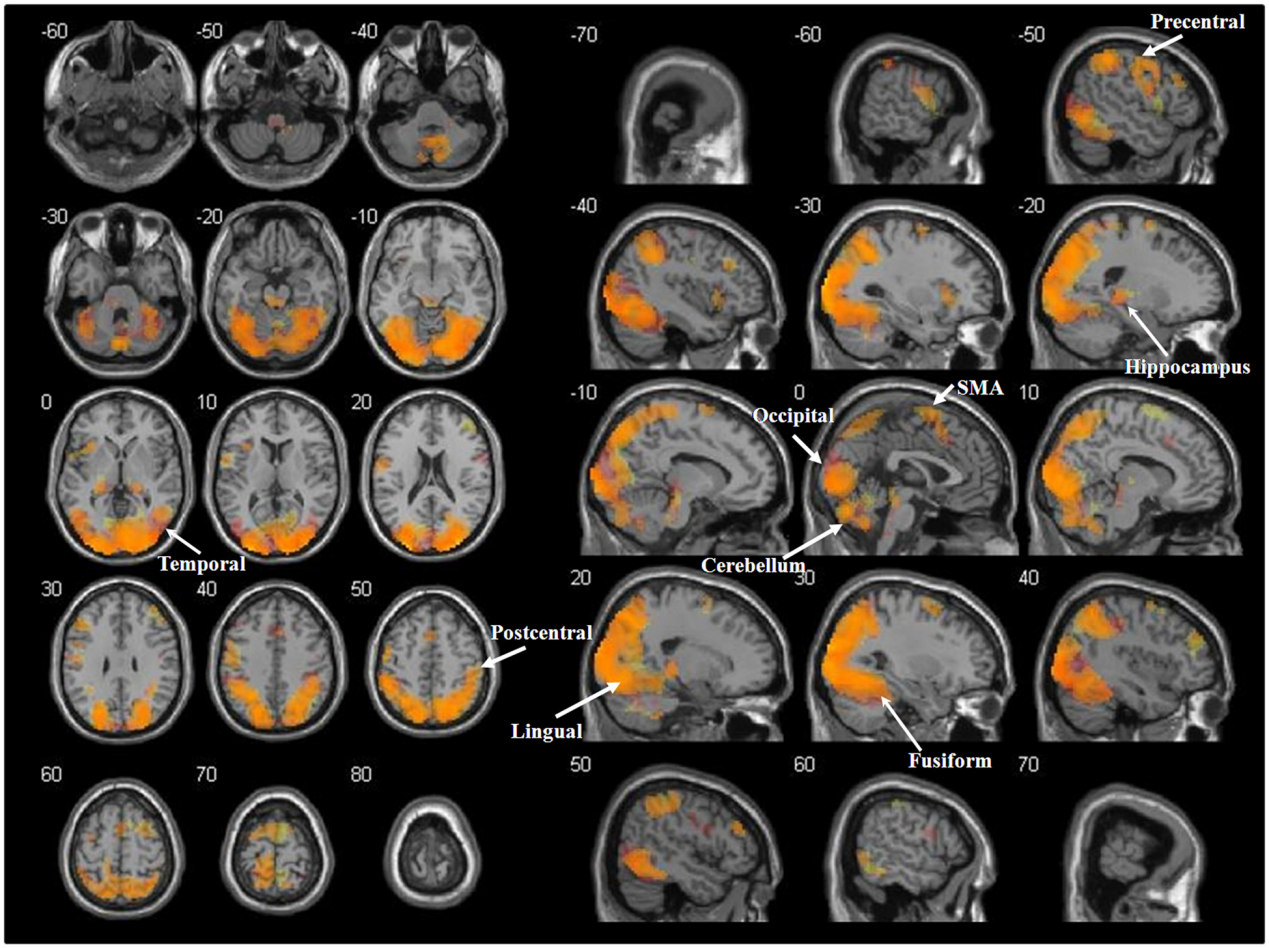
**Functional activations for the observation of arm movements and dot trajectories.** Cerebral regions involved in both the observation of arm movements and dot trajectories (orange) as revealed by conjunction analysis. The t-maps of AM > IB and DT > IB contrasts at the group level were thresholded (p < 0.05 at cluster-level, FWE corrected), binarised and multiplied voxel-wise with each other to identify common areas of activation. Areas activated only when arm movements were presented are shown in red, while areas activated only when dot trajectories were presented are shown in yellow. Activations are superimposed on the MNI single subject T1 template. The coordinates represented in the upper left corner of each section refer to the MNI stereotactic space.

The comparison of DTs observation with the IB revealed a large pattern of activations overlapping with the above described network. Consequently, the conjunction analysis identified significant activations in all the regions mentioned above, with medial frontal gyrus activated only in the left hemisphere and a right lateralisation of the cingulate gyrus and parahippocampal gyrus (Figure 3).

The differential analysis between AM and DT conditions (AM > DT) showed significant differences in occipital lobe, temporal lobe and cerebellum (Table 1). Finally, the reverse contrast (DT > AM) highlighted only small clusters of voxels in the occipital and temporal regions.

**Table 1:** Differential activations between conditions

**Contrast**	**Significant clusters**	**Brodmann areas**	**Size (voxels)**	**p**	**T**	**MNI coordinates**		
						**x**	**y**	**z**
**AM > DT**	Occipital_Mid_L, Fusiform_R, Calcarine_L, Fusiform_L, Occipital_Inf_R, Cuneus_R, Occipital_Inf_L, Occipital_Mid_R, Cuneus_L, Occipital_Sup_L, Lingual_L, Cerebelum_6_R, Occipital_Sup_R, Temporal_Mid_R, Cerebelum_6_L, Cerebelum_Crus1_L, Lingual_R, Temporal_Mid_L, Calcarine_R, Temporal_Inf_R, Cerebelum_Crus1_R, Temporal_Inf_L, Cerebelum_4_5_R, Parietal_Sup_R	19, 18, 37, 17, 39, 36, 20, 23	4008	0.000	14.65	−42	−88	4
**DT > AM**	Temporal_Mid_R, Temporal_Sup_R, Rolandic_Oper_R, SupraMarginal_R, Postcentral_R, Heschl_R	21, 22, 42, 43, 41, 40	167	0.000	6.21	57	−16	13
	Temporal_Inf_L, Temporal_Mid_L	37, 21, 22	54	0.061*	5.53	−54	−55	−11
	Angular_R, Parietal_Inf_R, Occipital_Mid_R, Occipital_Sup_R, Parietal_Sup_R	7, 40, 19, 39	88	0.009	4.84	36	−64	43
**AMT > DTT**	Precuneus_R, Precuneus_L, Parietal_Sup_R, Parietal_Sup_L	7	114	0.003	5.19	−6	−70	55
**DTT > AMT**	Occipital_Mid_L, Fusiform_R, Fusiform_L, Occipital_Mid_R, Occipital_Inf_R, Occipital_Inf_L, Occipital_Sup_R, Lingual_L, Cerebelum_6_R, Occipital_Sup_L, Lingual_R, Cerebelum_6_L, Cerebelum_Crus1_L, Cuneus_L, Cuneus_R, Calcarine_L, Temporal_Mid_R, Temporal_Mid_L, Temporal_Inf_R, Cerebelum_4_5_R, Cerebelum_Crus1_R	19, 18, 37, 39, 17, 7	1830	0.000	10.72	−42	−88	1
**CT > IT**	Caudate_R, Thalamus_L, Thalamus_R	---	184	0.000	6.93	12	5	16
	Postcentral_L, Precentral_L	4, 3, 6	57	0.069*	6.41	−51	−10	46
	Cerebelum_6_R, Fusiform_R, Lingual_R, Cerebelum_Crus1_R, Temporal_Inf_R	18, 19, 37	252	0.000	5.59	33	−58	−23
	Putamen_R, Amygdala_R, Olfactory_R, ParaHippocampal_R, Rectus_R, Frontal_Sup_Orb_R	34, 25, 47	80	0.021	5.35	15	8	−23
**AMCT > AMIT**	Frontal_Mid_R, Frontal_Inf_Tri_R	46, 10	76	0.021	6.67	45	47	4
	Postcentral_L, Precentral_L	4, 3, 6	55	0.069*	6.20	−51	−10	46
	Cerebelum_6_R, Fusiform_R, Cerebelum_Crus1_R, Temporal_Inf_R	37, 19	91	0.010	4.52	33	−58	−23
	Cingulum_Mid_L, Supp_Motor_Area_R, Supp_Motor_Area_L, Cingulum_Mid_R	24, 6, 31	74	0.024	4.00	0	−4	64
**DTCT > DTIT**	---	---	---	---	---	---	---	---
**IT > CT**	---	---	---	---	---	---	---	---
**AMIT > AMCT**	---	---	---	---	---	---	---	---
**DTIT > DTCT**	Occipital_Sup_L, Calcarine_L, Cuneus_L, Occipital_Mid_L, Lingual_L	18, 17, 19	189	0.000	7.24	−12	−99	10

For each contrast, all significant clusters (p < 0.05 at cluster-level, FWE corrected) are shown. The anatomical areas defined in the Automatic Anatomical Labeling (AAL) atlas [48] and Brodmann areas are listed for each cluster, ordered by decreasing number of voxels. T-score and MNI coordinates refer to the voxel with the peak value. Clusters with 0.05 ≤ p < 0.10 are also reported (marked with a star); these clusters were considered marginally significant.

### Representation of arm movements and dot trajectories and congruence analysis

A network of activations was observed when the representation of arm movements (AMT) was compared to the IB. Regions with significant activations included the occipital lobe (primary, secondary and associative visual cortex, BA 17, 18, 19), the parietal lobe (superior parietal lobules, BA 5, 7; right supramarginal gyrus, BA 40; angular gyrus, BA 39; postcentral gyrus, BA 3, 1, 2; precuneus, BA 7 medial; paracentral lobule, BA 3, 1, 2, 5 medial), the temporal lobe (superior, middle and inferior temporal gyrus, fusiform gyrus, BA 20, 21, 22, 37, 38), the frontal lobe (left precentral gyrus-primary motor area, BA 4; right SMA, BA 6; medial frontal gyrus, BA 8, 9; superior frontal gyrus, BA 4, 6, 8; middle frontal gyrus, BA 9; inferior frontal gyrus, BA 46, 47), the limbic lobe (hippocampus, parahippocampal gyrus, BA 27, 30), insula, cerebellum, basal ganglia (caudate, left putamen) and the thalamus.

**Figure 4:**
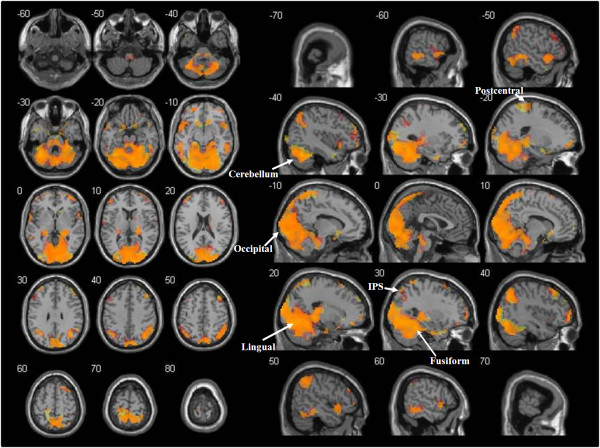
**Functional activations for the representation of arm movements and dot trajectories.** Cerebral regions involved in both the representation of arm movements and dot trajectories (orange) as revealed by conjunction analysis. The t-maps of AMT > IB and DTT > IB contrasts at the group level were thresholded (p < 0.05 at cluster-level, FWE corrected), binarised and multiplied voxel-wise with each other to identify common areas of activation. Areas activated only for arm movements’ representation are shown in red, while areas activated only for dot trajectories’ representation are shown in yellow. Activations are superimposed on the MNI single subject T1 template. The coordinates represented in the upper left corner of each section refer to the MNI stereotactic space.

Similar to the case of movement observation, an overlapping pattern of activations was found when comparing the representation of dot trajectories (DTT) to the IB, so that all the above cited regions were present in the conjunction analysis of the two contrasts (Figure 4).

The presentation of the target following arm movements was compared with the presentation of the target following dot trajectories, showing significant differences in the superior parietal cortex and in the precuneus (AMT > DTT, Table 1). The reverse contrast (DTT > AMT) revealed a large cluster of voxels in the occipital lobe, temporal lobe and cerebellum, with a similar pattern to that obtained with the AM > DT contrast.

When comparing the presentation of congruent and incongruent targets (CT > IT), significant differences were found in the right occipital lobe (secondary and associative visual cortex, BA 18, 19), left parietal (postcentral gyrus, BA 3), right temporal (inferior temporal gyrus and fusiform gyrus, BA 37, 34), left frontal (orbital cortex, BA 47; precentral gyrus-primary motor area, BA 4; SMA, BA 6; olfactory cortex, BA 25), and right limbic lobe (parahippocampal gyrus and amygdala). Significant activations were also found in cerebellum, caudate, putamen and in the thalamus. AMs mainly contributed to this result, as evident from the analysis of component contrasts. In particular, the AMCT > AMIT contrast revealed significant differences in the right occipital lobe (associative visual cortex, BA 19), left parietal lobe (postcentral gyrus, BA 3), right temporal lobe (inferior temporal gyrus, BA 37), bilateral frontal lobe (SMA, BA 6; precentral gyrus-primary motor area, BA 4; middle and inferior frontal gyrus, BA 46, 10), and bilateral limbic lobe (cingulate cortex, BA 24, 31); on the contrary, no cluster survived a p < 0.10 FWE corrected threshold when the DTCT > DTIT contrast was applied. In the reverse contrast (IT > CT) no cluster survived the corrected threshold. However, when component contrasts were analysed, while no cluster survived a p < 0.10 FWE threshold in the AMIT > AMCT contrast, the DTIT > DTCT contrast revealed significant activations in the left occipital cortex (primary, secondary and associative visual cortex, BA 17, 18, 19).

## Discussion

In the present study, an fMRI experiment was used to investigate the brain regions involved in a visual task adapted from the training with the InMotion2 Robot. The task required upper limb motor gesture recognition and activation of the cognitive representations of arm movements and dot trajectories, both presented on a screen. The results showed a caudo-rostral neuronal pathway. Common activations between upper limb gestures and dot trajectories were found with regard to both movement observation and retrieval. Furthermore, the activation of areas involved in higher level cognitive functions was associated with the processing of congruent trials more than with the incongruent ones.

### Observation of arm movements and dot trajectories

The pathway of activations found is consistent with previous studies [49-53] and points out that the neural correlates of movement are a property of the brain that emerges from a complex cortical-subcortical network. A significant bilateral activation was elicited during perception and recognition of arm movements, depicting a caudo-rostral pathway: visual perception (primary, secondary and associative visual cortex [54]), sensory integration [55], subsequent recognition of arm movement [56] and attention shifting [57,58] (posterior parietal cortex), spatial input processing [59] (inferior parietal cortex: supramarginal gyrus), re-mapping on the somatosensory and motor cortex (postcentral gyrus [60], precentral gyrus [61,62] and SMA [63]), storage in memory (hippocampus and parahippocampal gyrus, temporal areas [64-66] and dorsolateral pre-frontal cortex, see [67] for a review), motor control and motor learning (cerebellum [68], hippocampus and parahippocampal gyrus [69]). As far as the dorsolateral fronto-parietal activations are concerned, a similar pattern was observed when dot trajectories were presented, showing a wide overlapping of areas significantly activated during arm movement and dot trajectory processing, as revealed by a conjunction analysis. However, the larger activations in the lateral and medial occipital and temporal lobes in arm movement presentation with respect to dot trajectory trials, revealed by the AM > DT subtractive analysis, may only in part be explained by the visuo-perceptual differences among the two conditions. Actually, the more intense BOLD signal from lingual and fusiform gyri during the AM condition supports the specific role of these *loci* in biological movement processing and confirms previous fMRI data [30,31]. The wider cerebellar involvement in AM reveals the contribution of such a structure to motor activities (see also [30,32]). Interestingly, no difference was found in the motor and pre-motor areas.

### Representation of arm movements and dot trajectories

In each trial, following the presentation of the target, subjects were required to analyse the congruence between the observed arm movement or dot trajectory and the position of the target point. This implies retrieval from memory of the observed movement and imagery of the movement toward the target (both for human arm motor gestures, AMTs, and dot trajectories, DTTs). Results showed activation patterns consistent with data from previous studies [13-18]. The active *loci* corresponded to those found during movement observation, with the minor differences described in the Results section. Similar to the case of movement observation, a wide overlapping of activated areas for biological and non-biological trials was also found with regard to movement retrieval and imagery. This result was again confirmed by a conjunction analysis. However, more intense parietal cortical activity (superior parietal lobule) was found for AMT than for DTT. Together with premotor and prefrontal cortex, the superior parietal lobule is part of the cerebral network for working memory [70]. In the reverse contrast (DTT > AMT), the differential activations involved occipital and temporal lobes and the cerebellum, which were the same areas that were more activated during AM than DT observation.

While there is convincing evidence about the cerebral structures involved in biological motion processing, it is still not clear whether or not the processing of non-biological movements relies on the same structures. Previous studies have described the specific anatomical networks for the perception of biological movements, compared with non-biological ones [30-33]. However, other works have reported that observing upper limb movements carried out by humanoid robotic devices may generate the same fMRI pattern activated by the observation of a human arm [36-39]. Therefore, a possible explanation of the results provided by the present study would be that dot trajectories (i.e., abstract movements corresponding to the visual feedback in the InMotion2 Robot training) activate the same brain areas involved in the processing (recognition, retrieval and imagery) of the human arm motor gesture. In that case, our data would support the hypothesis that the non-biological feedback is processed in a manner similar to that for the natural motor gesture. As far as the dorsolateral fronto-parietal regions are concerned (in particular motor, pre-motor and sensorimotor areas), these results would enforce the idea that a biological input is not necessarily required [36-39], whereas significant differences mainly involve the lingual and fusiform gyri [30,31].

The fact that significant areas in the DTT > AMT comparison overlapped (with smaller extension and lower intensity) with those in which activations were stronger during arm movement presentation than during dot trajectory observation (AM > DT; Table 1), would suggest that when participants were seeing the target, and hence had to make the decision on the congruity of the dot trajectory previously seen, they mentally reconstructed the motor gesture that subtends such a trajectory. Therefore, in trials presenting dot trajectories, a more intense effort was required to reconstruct the human gesture generating the corresponding non-biological motion. On the contrary, in case of previous presentation of human arm movements, subjects did not need to actively reconstruct the gesture, since a stored trace was still available in working memory. However, a possible objection could be that such a result may also be related to the characteristics of our experimental design. In the event-related task that was implemented in this study, the presentations of AM and DT trials were randomised, which could have facilitated a strategy based on the recall of the corresponding motor gesture even in case of dot trajectory presentation. In summary, from a rehabilitative point of view, we may speculate that inducing patients with hemiplegia to mentally reconstruct the gestures by requiring an active analysis of the goal of the action, might increase the cerebral haemodynamic response from the network involved in movement processing, with respect to passive stimulation only. This argument implies that the motion analysis of inanimate objects could be more effective than just observing a gesture or requiring the immediate action repetition.

### Processing of congruence

Results from the analysis of congruent vs. incongruent trials revealed more intense neural activity in a few cortical areas for congruent conditions. *Post hoc* analyses demonstrated that such an effect should be ascribed to trials with arm movement presentation. Indeed, in this condition we found greater activity in a network including bilateral cingulate cortex, right inferior and middle frontal gyrus that are involved in the go-signal and in decision control [71-73], SMA and, with a marginal significance, left primary motor and sensorimotor areas that are involved in perception of limb movements and motor imagery [52,62]. These differences could depend on the task demand characteristics, namely on the fact that participants were asked to mentally count the number of overall congruent conditions. For that reason, congruent information was more relevant for decision with respect to the incongruent one and participants may have repeated and reinforced the mental reconstruction of the congruent gesture to avoid mistakes. Subjects were instructed to count according to the following reasoning: “If congruent trial (regardless biological or non-biological), then update the count”. No reason allows to suppose that they used different strategies for biological and non-biological trials, since congruent trials were counted jointly. The mental counting procedure could account for activation within and around the IPS, since such area is commonly associated with number processing [74-77], and for Broca’s area activation, as far as silent speech production is concerned. With the exclusion of these two areas, there is no reason to ascribe significant cortical activations to a counting process.

The role played by the knowledge of results provided by the feedback during robotic training deserves further consideration. In the fMRI task, the target point was shown after the end of movement observation and it was not superimposed on the motor gesture or dot trajectory. Literature on motor learning suggests that a delayed feedback (i.e., a feedback provided at the end of the action), similar to the one used in our study, increases the processing of the features of performance and promotes a more stable learning than an instantaneous feedback, like the one provided during the InMotion2 Robot training [78]. While this paper addresses the issue of the biological or non-biological nature of the visual feedback, the different effectiveness of continuous vs. delayed feedback during robotic therapy remains unclear.

## Conclusions

A visual fMRI task was used to identify the neural pathway associated with the visual processes involved in upper limb motor training performed with the InMotion2 Robot. This study investigated the suitability of non-biological movement presentation, with respect to human movement observation, in activating brain networks for motor processing. Results from healthy adult subjects would support the appropriateness of the visual feedback (movement of a dot) during robotic treatment, while the task does not address whether a continuous feedback is more or less efficient than a feedback based on delayed knowledge of results. However, due to the nature of the task, this study does not take into account some relevant contributions that affect motor control such as motor learning related to the haptic feedback and the aspects of motor execution provided by robotic training. Moreover, the dissociation between the processing of arm movements and abstract object trajectories cannot be fully discarded since its absence in the results could be due to the characteristics of our task, which, similar to what happens during robotic training, may induce the assimilation of strategies for upper limb movement and dot trajectory processing.

## Abbreviations

AM, Observation of an arm movement; AMCT, Presentation of a congruent target point following an arm movement; AMIT, Presentation of an incongruent target point following an arm movement; AMT, Presentation of (congruent or incongruent) target points following arm movements (also referred to as “representation of arm movements”); BA, Brodmann area; CT, Presentation of congruent targets (following arm movements or dot trajectories); DT, Observation of a dot trajectory; DTCT, Presentation of a congruent target point following a dot trajectory; DTIT, Presentation of an incongruent target point following a dot trajectory; DTT, Presentation of (congruent or incongruent) target points following dot trajectories (also referred to as “representation of dot trajectories”); EPI, Echo-planar imaging; fMRI, Functional magnetic resonance imaging; FWE, Family-wise error; IB, Implicit baseline; IPS, Intraparietal sulcus; IT, Presentation of incongruent targets (following arm movements or dot trajectories); KO, Kinetic occipital area; MNI, Montréal neurological institute; SMA, Supplementary motor area; SPM, Statistical parametric map; STS, Superior temporal sulcus.

## Competing interests

The authors have not competing interests as defined by the BioMed Central Publishing Group, or other interests that may influence results and discussion reported in this study.

## Authors’ contributions

FN contributed to conception and design, test conduction, data acquisition, data analysis and writing. SG contributed to conception and design, test conduction, data acquisition, interpretation of data and writing. CG contributed to test conduction, interpretation of data and writing. MP contributed to conception and design, test conduction, interpretation of data and writing. VC, PC, TD, EC participated in design, acquisition of founding and coordination of the research. All authors read and approved the final manuscript.
